# Effectiveness and safety of ferric carboxymaltose treatment in children and adolescents with inflammatory bowel disease and other gastrointestinal diseases

**DOI:** 10.1186/1471-230X-14-184

**Published:** 2014-10-17

**Authors:** Martin W Laass, Simon Straub, Suki Chainey, Garth Virgin, Timothy Cushway

**Affiliations:** Department of Pediatrics, University Hospital Carl Gustav Carus Dresden, Technische Universität Dresden, Fetscherstraβe 74, Dresden, 01307 Germany; Statistics Department, Vifor Pharma Ltd, Flughofstrasse 61, Glattbrugg, CH-8152 Switzerland; Medical Department, Vifor Pharma Ltd, Flughofstrasse 61, Glattbrugg, CH-8152 Switzerland

**Keywords:** Pediatric, IBD, Intravenous iron, Ferric carboxymaltose, Iron deficiency, Anemia

## Abstract

**Background:**

The treatment of iron deficiency anemia in children with inflammatory bowel disease is a particular challenge and often insufficient. Absorption of orally given iron may be impaired by intestinal inflammation and treatment with oral iron may aggravate intestinal inflammation. This retrospective study is the first to describe the use of intravenous ferric carboxymaltose (FCM) in the pediatric setting.

**Methods:**

All subjects who had received at least one dose of FCM intravenously in the observation period were included in this analysis with data collected for up to 3 months post last FCM dose.

**Results:**

In total, 72 children between 0 and 18 years with underlying gastrointestinal disorders had been treated for concomitant iron deficiency anemia. The majority of patients had Crohn’s disease (40.3%) or ulcerative colitis (30.5%). The total number of FCM administrations was 147, the mean number per patient was 2.0 and the mean cumulative dose 821 mg iron (median single dose: 500 mg; max. 1000 mg). Post administration of FCM, correction of iron deficiency anemia was observed with improved mean hemoglobin levels from 9.5 g/dL at baseline to 11.9 g/dL within 5–12 weeks. Decreases in white cell count, platelets and C-reactive protein were observed post FCM, potentially suggesting reduced inflammation with iron repletion. Three subjects reported mild adverse drug reactions related to FCM; two of these were considered to be potentially related to long duration of administration and to high volume of saline solution for dilution. As such, the method of administration was amended to have a maximum infusion time of 60 minutes and dilution with less than or equal to 100 mL saline solution.

**Conclusions:**

Overall FCM was well tolerated in this pediatric population and appeared to be effective in correcting iron deficiency anemia. We cannot exclude that the correction of iron deficiency anaemia is in some part due to the treatment of the underlying disease and not related to the iron supplementation only.

**Electronic supplementary material:**

The online version of this article (doi:10.1186/1471-230X-14-184) contains supplementary material, which is available to authorized users.

## Background

Children with inflammatory bowel disease (IBD) are at increased risk for iron deficiency for multiple reasons: increased requirements during growth, insufficient dietary intake, decreased absorption of dietary iron and intestinal blood loss. In a recent study 75% of children with IBD were anaemic at diagnosis, and 90% of children with Crohn’s disease and 95% of children with ulcerative colitis had iron deficiency at diagnosis. Even at two years follow-up, 30% of children with IBD were anaemic and 70% of children with Crohn’s disease and 65% with ulcerative colitis were still iron deficient [1]. As such, anemia is more common in children than in older IBD patients [2].

In IBD usually two forms of anemia coexist: iron-deficiency anemia (IDA) and anemia of chronic disease. Therefore, careful evaluation of iron status, including laboratory parameters like hemoglobin (Hb), mean corpuscular volume, serum ferritin, transferrin saturation, is mandatory to diagnose and treat IDA early during the course of disease. For the correct assessment of iron status, parameters of inflammation such as C-reactive protein must also be taken into consideration. Similar to adult settings, caution is recommended with interpretation of iron stores based on serum ferritin, as this acute phase reactant may be normal or elevated even in the presence of iron deficiency. For this reason, ferritin levels up to 100 μg/L may still be consistent with iron deficiency, if there are indications for inflammation [3, 4].

IDA may negatively impact quality of life due to increased chronic fatigue and reduced energy metabolism, these being due to the role of iron in myoglobin as well as numerous other metabolic and even cognitive functions [5]. Hence, IDA warrants early and appropriate intervention to relieve not only acute symptoms of iron deficiency but also the longer-term potential consequences. The treatment of IDA is a particular challenge in the IBD population; on the one hand iron absorption may be impaired by intestinal inflammation, whilst on the other hand oral iron treatment may worsen IBD symptoms by aggravating intestinal inflammation [6].

There is a high intolerance and non-adherence to oral iron in children and adults due to the side effects like nausea, abdominal pain and constipation [7]. With intravenous preparations intestinal absorption can be bypassed and iron replacement can be achieved with one or two doses of the newer high-dose preparations. Although the rationale is compelling, unfortunately in the pediatric setting very few studies have examined the role of intravenous iron for correction of IDA. In contrast, the role of intravenous iron is well established as a therapeutic option in numerous areas, including IBD, in the adult population [8].

There are a number of different intravenous iron formulations: high and low molecular weight iron dextran, iron sucrose, ferric gluconate, ferric carboxymaltose and ferumoxytol. As intravenous iron therapies have different physico-chemical properties, the administration may require more frequent lower individual doses (e.g. intravenous iron sucrose or gluconate) whereas more stable preparations (e.g. iron dextran or ferric carboxymaltose) can be given as large single intravenous doses. Concerns regarding the safety of intravenous iron preparations refer particularly to high molecular weight dextran formulations, which have an increased risk of anaphylaxis resulting from anti-dextran antibodies [9]. Newer intravenous iron drugs such as iron sucrose and ferric carboxymaltose (FCM) appear to have better safety profiles [10, 11].

FCM has been studied in numerous therapeutic areas including IBD [11–13] and has almost 1.2 million patient years of exposure as of June 2013 (based on an average dosing of 1,350 mg iron per patient) [14]. The product was first approved in Europe in 2007 for the correction of iron deficiency in patients over the age of 14 years. FCM is dextran-free and may be administered in large single doses up to 1000 mg iron either as an infusion (limited at 20 mg iron per kg body weight) or injection (limited at 15 mg iron per kg body weight) and does not require a test dose.

To date, no data have been published on the use of FCM in the pediatric population and hence this retrospective analysis is the first study to provide initial experiences in treatment of IDA in children with IBD and other gastrointestinal diseases associated with IDA.

## Methods

All patients 18 years and younger treated with FCM for IDA during the period 1 September 2008 and 30 April 2013 at the University Hospital for Children and Adolescents, Dresden, Germany were considered for inclusion.

Anemia was defined as Hb below the lower reference value (according to age and gender) of the local laboratory (Additional file 1: Table S1). Anemia was classified as IDA when the following values were below the lower reference value of the laboratory: mean corpuscular volume of the erythrocytes, transferrin saturation and ferritin (<20 μg/L). Anemia was also classified as IDA when the soluble transferrin receptor or the soluble transferrin receptor/log ferritin ratio was elevated (Additional file 1: Table S1). When parameters of inflammation were present (e.g. clearly elevated C-reactive protein, erythrocyte sedimentation rate), ferritin values up to 100 μg/L were regarded consistent with IDA.

Patients receiving at least one dose of FCM (Ferinject®; Vifor Pharma, St Gallen, Switzerland) had data transcribed to a case report form and were included in this retrospective analysis. To avoid selection bias, all patients treated with FCM during the period, irrespective of diagnosis or outcome, were documented.

The study was approved by the local ethics committee of the Technische Universität Dresden (EK 411122010). Ethical permission for collection of anonymous data was granted prior to transcription from patient medical records to a case report form. No interventional procedures or changes to current practice of patient management were required and all treatment decisions had been made prior to the initiation of this observational study. In addition, parents of children younger than 14 years at time of receiving FCM gave informed consent for the off-label use of FCM (as the product was approved only for use in patients 14 years or older at the time of administration).

Collected data included demography, significant medical history and disease status. Additionally, information on FCM use including date and dose administered as well as route was recorded. Iron requirements had been determined using the Ganzoni formula at the time of treatment [15, 16]. Standard laboratory parameters from routine medical practice along with concomitant medications taken three months prior and three months post last FCM dose were recorded as well as adverse events documented up to three months post drug administration.

Laboratory parameters included Hb, mean corpuscular volume, hematocrit, mean corpuscular Hb, mean corpuscular Hb concentration, serum ferritin level, transferrin saturation, soluble transferrin receptor, soluble transferrin receptor/ log ferritin ratio, reticulocytes, reticulocyte Hb, red blood cell distribution width, white blood cell count, absolute neutrophil count, platelets, C-reactive protein, aspartate transaminase, alanine transaminase, gamma glutamyl transferase and phosphate. Due to the retrospective nature of the study and limited sample size, laboratory parameters were grouped in time periods. This included pre-FCM (any value available from 12 weeks prior to first dose), day of first FCM administration (which is generally referred to as baseline unless missing, in which cases values from pre-FCM were used as baseline) and then in ranges of weeks, specifically weeks 0–2, 2–4, 5–8 and 9–12, in which each range included values from within the specified period (Weeks 0–2 comprise days 1 to 14 and weeks 2–4 comprise days 15 to 28).

The statistical analysis was descriptive only. Continuous data were summarised by mean, standard deviation, median, lower and upper quartiles, minimum and maximum values. Analysis of variance (ANOVA) or analysis of covariance (ANCOVA) was used for comparison. Categorical data were summarised by the number and percentage of subjects in each category.

## Results

In total, 72 children and adolescents with underlying gastrointestinal disease and IDA were treated with FCM during the period of 1 September 2008 and 30 April 2013. Similar numbers of female and male subjects were treated with a mean age of 12.7 years (range: 0 to 18 years). The youngest patient was an eleven months old infant after liver transplantation for biliary atresia. The majority had Crohn’s disease (40.3%) or ulcerative colitis (30.5%) and were receiving concomitant immunosuppressive therapy for their underlying disease. In all cases, the reason for initiating FCM was IDA and just over half of the patients had previous oral iron use. Demographics and additional baseline details are further described in Table 1. 37/72 (51.3%) of our patients were younger than 14 years when they received their first dose of FCM.

**Table 1:** **Patient characteristics and concomitant medication**

Total n	72
**Gender, n (%)**	
Male	35 (48.6)
Female	37 (51.4)
**Age, years**	
Mean (SD)	11.8 (4.86)
Min, max	0, 18
Median	13.5
Interquartile range	8, 15.5
**Previous oral iron use, n (%)**	39 (54.2)
**Medical history, n (%)**	
Crohn’s disease	29 (40.3)
Ulcerative colitis	22 (30.5)
Celiac disease	4 (5.6)
Gastroesophageal reflux disease	2 (2.8)
Helicobacter pylori gastritis	2 (2.8)
Oesophageal varices bleeding	2 (2.8)
Chronic diarrhoea	1 (1.4)
Hyper-IgM syndrome	1 (1.4)
Intestinal vascular malformation	1 (1.4)
Ulcus ventriculi	1 (1.4)
Others	7 (9.7)
**Concomitant medication, n (%)**	
**Any medication**	**69 (95.8)**
Prednisolone	30 (41.7)
Azathioprine	29 (40.3)
Mesalazine	34 (47.2)
Omeprazole	31 (43.1)
Ferrous glycine sulphate	15 (20.8)
Metronidazole	10 (13.9)
Infliximab	9 (12.5)
Escherichia coli Nissle 1917	9 (12.5)
Cyclosporine	5 (6.9)

Most often used concomitant medication taken at any time during the observation period (i.e. 3 months prior and 3 months post administration of FCM). SD: Standard deviation.

The total number of FCM administrations was 147 and the mean number per patient was 2.0. The mean cumulative dose of intravenous FCM was 821 mg iron given in two single infusions (median dose: 500 mg iron diluted in 100 mL saline over 66 minutes, max. dose: 1000 mg). On average, patients received iron dosing at 12 mg/kg body weight with a maximum single administration of 32 mg iron per kg body weight in a 9 year-old girl weighing 31 kg. Whilst the majority of individual doses were administered at levels <15 mg/kg, 16 patients received at least 1 dose ≥15 mg/kg and 7 received a dose >20 mg/kg. Calculated baseline iron requirements (using the Ganzoni formula) ranged from 100 to 1700 mg iron and closely matched the doses given to patients. Details are described in Table 2. During administration of FCM and until two hours thereafter, patient’s oxygen saturation, heart rate and blood pressure were closely monitored.

**Figure 1:**
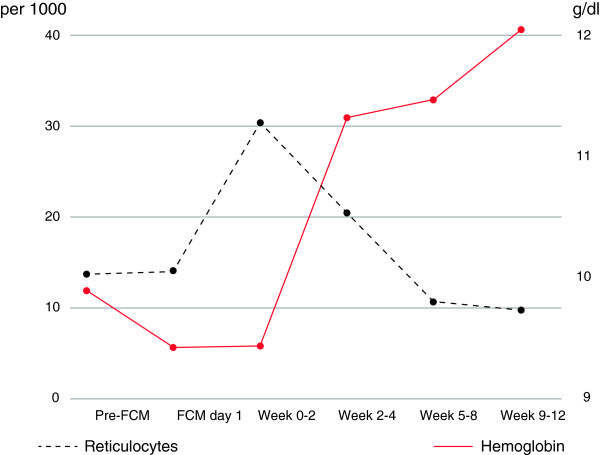
**Time course of reticulocytes and hemoglobin.** Given are reticulocytes per 1000 erythrocytes (left axis, black line) and hemoglobin in g/dL (right axis, red line). FCM day 1 refers to day of first FCM administration, but blood for assessment of laboratory parameters was drawn before FCM administration. This is referred to as baseline (unless values from this day were missing, in which cases Pre-FCM values were used). Additional FCM doses may have been administered after this to achieve iron repletion. Weeks 0–2 comprise days 1 to 14 and weeks 2–4 comprise days 15 to 28.

**Figure 2:**
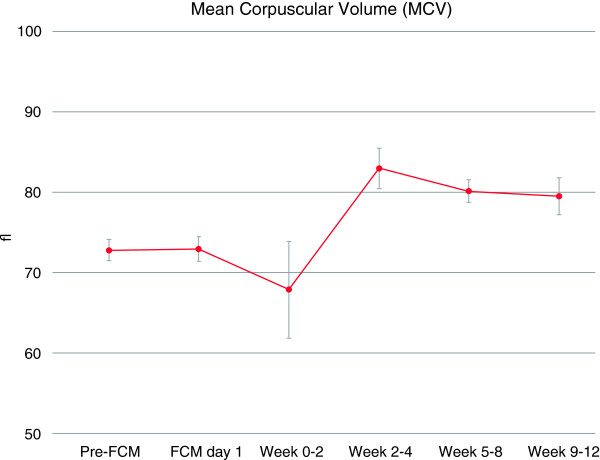
**Time course of mean corpuscular volume over observational period.** Given are mean values in femtoliter (fL) and standard errors. FCM day 1 refers to day of first FCM administration, but blood for assessment of laboratory parameters was drawn before FCM administration. This is referred to as baseline (unless values from this day were missing, in which cases Pre-FCM values were used). Additional FCM doses may have been administered after this to achieve iron repletion. Weeks 0–2 comprise days 1 to 14 and weeks 2–4 comprise days 15 to 28.

**Figure 3:**
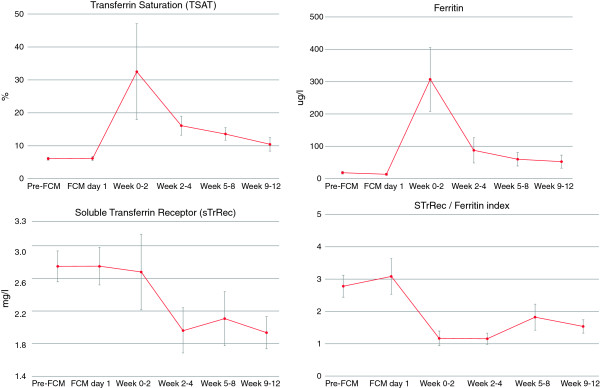
**Time course of iron parameters.** Given are mean value and standard errors for serum concentration of transferrin saturation (in %), ferritin (in μg/L) and soluble transferrin receptor (in mg/L) and for soluble transferrin receptor/ log ferritin ratio during the course of the observation period. FCM day 1 refers to day of first FCM administration, but blood for assessment of laboratory parameters was drawn before FCM administration. This is referred to as baseline (unless values from this day were missing, in which cases Pre-FCM values were used). Additional FCM doses may have been administered after this to achieve iron repletion. Weeks 0–2 comprise days 1 to 14 and weeks 2–4 comprise days 15 to 28.

Post administration of FCM, correction of IDA was observed with early reticulocyte level increases from baseline up until week 4. This translated into improved Hb concentration with values evolving from a baseline of 9.5 g/dL (5.9 mmol/L) to 11.4 g/dL (7.1 mmol/L) within 5–8 weeks and stabilizing at 11.9 g/dL (7.4 mmol/L) by week 9–12 (Figure 1). Similar to Hb, the mean corpuscular volume levels were low at baseline indicating microcytic anemia and were seen to improve rapidly post FCM administration and stabilize for the duration of the observational period (Figure 2). The measured iron parameters demonstrated repletion of iron with a peak at approximately 2 weeks post administration that thereafter plateaued with values in the normal or target range in most patients (Figure 3).

**Table 2:** **Summary of ferric carboxymaltose (FCM) use and iron dosing**

Total n	72
**Total cumulative iron dose [mg]**	
Mean (SD)	820.8 (741.04)
Median	500.0
Min, max	50, 3150
**Amount of iron per dose [mg]**	
Mean (SD)	406.7 (203.07)
Median	500.0
Min, max	50, 1000
**Total number of administrations**	147
**Total number of administrations per patient**	
Mean (SD)	2.0 (1.75)
Median	1.0
**Number of administrations per patient, n (%)**	
1	39 (54.2)
2	17 (23.6)
3	8 (11.1)
4	3 (4.2)
7	2 (2.8)
8	3 (4.2)

SD: Standard deviation.

**Figure 4:**
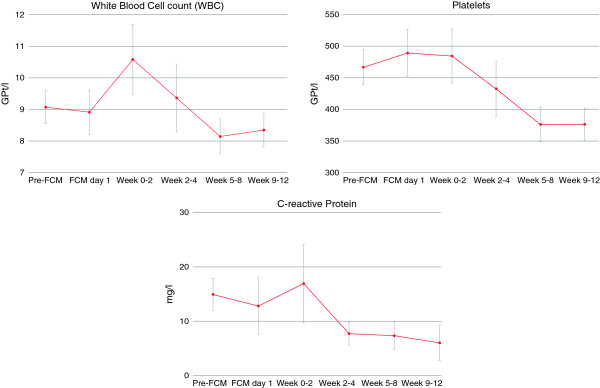
**Time course of white blood cell count, platelets and C-reactive protein.** White blood cell count and platelets are given in Gpt/L and C-reactive protein (CRP) in mg/L. Given are mean values and standard errors.

In addition to the above changes in relation to Hb and iron parameters, decreases in white cell count, platelets and C-reactive protein were also observed post FCM administration. Mean white cell count was observed to decrease from a value of 8.6 Gpt/L at baseline (prior to FCM administration) to a value of 7.5 Gpt/L between weeks 5–8, whilst platelet levels decreased from 487 Gpt/L to values of 376 GPt/L by weeks 5–8 and remained at this level thereafter. Decreases were also observed for mean C-reactive protein levels with values declining from an initial 12.8 mg/L at baseline to values of 7.4 and 6.0 mg/L by weeks 5–8 and weeks 9–12 respectively (Figure 4).

Most patients had values within normal range for aspartate transaminase (AST), alanine transaminase (ALT), gamma glutamyl transferase (GGT) and phosphate with no excursions that led to a need to discontinue FCM or delay dosing.

The majority of subjects (69 of 72, 95.8%) received concomitant medications during the 3 months following last dose of FCM. The most frequently reported concomitant medications were mesalazine, omeprazole and prednisolone. These medications are consistent with treating the underlying aetiology of the included population.

In relation to safety, 3 subjects reported adverse drug reactions to FCM. The first event was seen in a 17-year-old male who experienced mild urticaria on the first day of treatment with FCM, which resolved without treatment the following day. He received two further infusions of FCM and no further adverse drug events were recorded with subsequent doses. The second event occurred in a 5-year-old male who experienced mild oedema of the palms and fingers of both hands on the first day of treatment with FCM. After antihistaminic treatment (with oral dimetinden maleat) symptoms resolved the same day. The subject received a total of three infusions of FCM and no reaction was observed with the other administrations. Both mild adverse drug reactions were considered potentially related to long duration of administration and high volume of saline for dilution, which was not in accordance with the manufacturer’s instruction to avoid dilution to iron concentrations below 2 mg/mL. After altering the method of administration to a dilution with 100 mL saline or less and an infusion time not exceeding 60 minutes, only one further adverse drug reaction occurred. The third event occurred in a 15-year old female who experienced mild urticaria on the first and only day of treatment with FCM. Concomitant medication (dimetindene maleate) was administered and the event resolved on the same day. The event was considered related to FCM by the investigator and the subject only received one infusion of FCM. No serious or severe adverse drug events were reported in the treated patients.

## Discussion

The rationale for adopting the use of ferric carboxymaltose in our pediatric patients was to avoid poorly tolerated oral iron therapy and permit higher single doses of iron to be administered in shorter time periods with less overall numbers of infusions. The large pool of published clinical data, including studies performed in adult IBD patients, helped support our decision for implementing this product in our clinical practice. Until then we had prescribed oral iron preparations, used intravenous ferrous gluconate - often requiring multiple infusions - or tolerated IDA in our pediatric IBD patients. After having treated patients with FCM for nearly 5 years, we felt it prudent to systematically review the outcomes of this IDA management strategy.

The use of FCM did indeed permit fewer infusions on a per patient basis, with a median of one infusion per patient to deliver 500 mg iron (mean dose was 821 mg iron administered in two infusions). The effectiveness of dosing to correct iron levels was assessed by serum ferritin (providing insight on iron stores), transferrin saturation (providing insight on circulating iron available for use), soluble transferrin receptor and red blood cell parameters. Serum ferritin levels were returned to ‘normal’ targets by the end of the observation period; however, the transferrin saturation initially increased (at weeks 2–8) to near target ranges, but were then seen to decrease again by week 12 back to near baseline levels. This may reflect immediate iron utilization in the bone marrow, underestimation of total calculated iron deficit and/or ongoing losses which suggest a need for additional iron to avoid recurrent IDA.

In terms of anemia treatment, a secondary efficacy parameter, a rapid increase in the reticulocyte count was observed within 2 weeks of iron administration using FCM and translated into anemia correction already at approximately 4 weeks. Over the observational period, the Hb levels were seen to evolve from a pre-treatment mean of 9.5 g/dL (5.9 mmol/L) to an acceptable 11.4 g/dL (7.1 mmol/L) by 5–8 weeks and achieve acceptable concentrations of 11.9 g/dL (7.4 mmol/L) by weeks 9–12.

In recent studies of FCM use in an adult IBD population, similar results were observed, namely the correction of Hb to acceptable levels, albeit with a large proportion of patients not achieving or maintaining appropriate iron repletion. In this study the authors concluded that sufficient doses of iron may not have been given and that perhaps the current guidelines are not entirely suitable for optimal anemia management [13]. In the follow-up study to assess maintenance therapy (beyond initial iron deficiency correction) it was confirmed that anemia recurrence could be avoided in a large percentage of patients by means of continued iron management based on decreased serum ferritin values [17]. Whilst not currently our routine practice nor part of this observational study, such a maintenance strategy may warrant further investigation and provide rationale for more routine monitoring of iron parameters in our patients.

Although anemia and iron deficiency are highly prevalent in pediatric patients with IBD, focus of treatment lies often only on intestinal inflammation, which results in a slow hematological recovery [18]. Further to anemia management, the administration of iron also appeared to result in decreases respectively normalization of white cell count, platelets and C-reactive protein. These observations might suggest a role of iron therapy also in reducing inflammation and normalization of secondary thrombocytosis in IBD, however should be interpreted with caution, as medication changes were not captured as part of this study. As the majority of our patients received concomitant, mostly immunosuppressive, medication, such findings need to be confirmed in appropriately designed clinical studies. However, similar reductions have been seen in other studies using FCM, where a decrease of platelets was observed independent of inflammation markers such as C-reactive protein [19]. Therefore, decreases in platelet count in our study may reflect normalized bone marrow activity rather than reduced inflammation.

As with all intravenous iron preparations, there was a potential for side effects and hence tolerability was closely monitored during administrations. When initially using the medication, we adopted what we believed would be a safer approach and diluted FCM beyond recommendations provided in the product label and administered the product very slowly via infusion. However, this may have resulted in two events seen that were considered related to the study drug, namely a case of urticaria and one of mild oedema of the palms and fingers of both hands. As described above, these events were considered to be related to (a) long duration of drug administration and (b) high volume of saline for dilution of FCM. The exact mechanism is not known but may have been secondary to excess dilution leading to less stable complex (as the stability is dependent on the concentration equilibrium of the sodium hydroxide, iron [as iron(III)-hydroxide] and carboxymaltose). The method of administration was consequently amended to have a maximum infusion time of 60 minutes and dilution with 100 mL saline or less.

The authors recognize the limitations of this retrospective observational study, for example that a comparator arm is lacking which would permit any claims of efficacy or safety in comparison to alternative options. Nonetheless, to minimize bias, all patients treated with FCM during the period were documented, regardless of outcome. The dosing and administration were per routine clinical practice and not per any pre-specified protocol. Patients received doses at the discretion of the treating physician and as such, this study provides insights about product tolerance and effectiveness in day-to-day clinical practice, something not so easily observed in controlled studies.

Due to the observational nature of this study, we cannot confirm if the correction of iron deficiency anaemia is related to the iron supplementation only or also in some part to the treatment of the underlying active disease. This is compounded due to the limitation within our data collection which did not include disease activity at the time of iron treatment and hence we are unable to distinguish clearly between patients with active disease and those in remission. Hence further randomised controlled studies may be required to confirm the true impact of the iron therapy.

These initial experiences using FCM suggest that this non-dextran based intravenous iron may be well tolerated and suitable for use in the correction of iron deficiency anemia also in pediatric patients, especially for those with IBD. The option to give higher dose single injections or infusions may be of considerable benefit in the management of care in this special population. Currently, the product is approved only for use in patients 14 years of age or older and hence, further clinical studies are warranted to confirm the above observations.

## Conclusions

Overall FCM appeared to be safe and effective in correcting iron deficiency anemia in this pediatric population.

## Electronic supplementary material

Additional file 1: Table S1: Reference ranges for blood tests - used in the study - at various ages. (PDF 54 KB)

## Abbreviations

FCM: Ferric carboxymaltose
Hb: Hemoglobin
IBD: Inflammatory bowel disease
IDA: Iron deficiency anemia.

## References

[1] Wiskin AE, Fleming BJ, Wootton SA, Beattie RM (2012). Anaemia and iron deficiency in children with inflammatory bowel disease. J Crohns Colitis.

[2] Goodhand JR, Kamperidis N, Rao A, Laskaratos F, McDermott A, Wahed M, Naik S, Croft NM, Lindsay JO, Sanderson IR, Rampton DS (2012). Prevalence and management of anemia in children, adolescents, and adults with inflammatory bowel disease. Inflamm Bowel Dis.

[3] Rufo PA, Denson LA, Sylvester FA, Szigethy E, Sathya P, Lu Y, Wahbeh GT, Sena LM, Faubion WA (2012). Health supervision in the management of children and adolescents with IBD: NASPGHAN recommendations. J Pediatr Gastroenterol Nutr.

[4] Gasche C, Berstad A, Befrits R, Beglinger C, Dignass A, Erichsen K, Gomollon F, Hjortswang H, Koutroubakis I, Kulnigg S, Oldenburg B, Rampton D, Schroeder O, Stein J, Travis S, Van Assche G (2007). Guidelines on the diagnosis and management of iron deficiency and anemia in inflammatory bowel diseases. Inflamm Bowel Dis.

[5] Lozoff B, Beard J, Connor J, Barbara F, Georgieff M, Schallert T (2006). Long-lasting neural and behavioral effects of iron deficiency in infancy. Nutr Rev.

[6] Carrier J, Aghdassi E, Platt I, Cullen J, Allard JP (2001). Effect of oral iron supplementation on oxidative stress and colonic inflammation in rats with induced colitis. Aliment Pharmacol Ther.

[7] Schröder O, Mickisch O, Seidler U, de Weerth A, Dignass AU, Herfarth H, Reinshagen M, Schreiber S, Junge U, Schrott M, Stein J (2005). Intravenous iron sucrose versus oral iron supplementation for the treatment of iron deficiency anemia in patients with inflammatory bowel disease - a randomized, controlled, open-label, multicenter study. Am J Gastroenterol.

[8] Lee TW, Kolber MR, Fedorak RN, van Zanten SV (2012). Iron replacement therapy in inflammatory bowel disease patients with iron deficiency anemia: a systematic review and meta-analysis. J Crohns Colitis.

[9] Thayu M, Mamula P (2005). Treatment of iron deficiency anemia in pediatric inflammatory bowel disease. Curr Treat Options Gastroenterol.

[10] Kent AJ, Blackwell VJ, Travis SP (2012). What is the optimal treatment for anemia in inflammatory bowel disease?. Curr Drug Deliv.

[11] Beigel F, Löhr B, Laubender RP, Tillack C, Schnitzler F, Breiteneicher S, Weidinger M, Göke B, Seiderer J, Ochsenkühn T, Brand S (2012). Iron status and analysis of efficacy and safety of ferric carboxymaltose treatment in patients with inflammatory bowel disease. Digestion.

[12] Kulnigg S, Stoinov S, Simanenkov V, Dudar LV, Karnafel W, Garcia LC, Sambuelli AM, D’Haens G, Gasche C (2008). A novel intravenous iron formulation for treatment of anemia in inflammatory bowel disease: the ferric carboxymaltose (FERINJECT) randomized controlled trial. Am J Gastroenterol.

[13] Evstatiev R, Marteau P, Iqbal T, Khalif IL, Stein J, Bokemeyer B, Chopey IV, Gutzwiller FS, Riopel L, Gasche C, FERGI Study Group (2011). FERGIcor, a randomized controlled trial on ferric carboxymaltose for iron deficiency anemia in inflammatory bowel disease. Gastroenterology.

[14] Periodic Safety Update Report for Ferinject: *Vifor Pharma (PSUR PVZ-VIT45/E09) dated 16-Aug-2013*. Submitted to the MHRA, UK being the Reference Member State for the registration of Ferinject

[15] Ganzoni AM (1970). Intravenous iron dextran: therapeutic and experimental possibilities. Schweiz Med Wochenschr.

[16] Lyseng-Williamson and Keating (2009). Ferric Carboxymaltose - A Review of its Use in Iron-Deficiency Anaemia. Drugs.

[17] Evstatiev R, Alexeeva O, Bokemeyer B, Chopey I, Felder M, Gudehus M, Iqbal T, Khalif I, Marteau P, Stein J, Gasche C, FERGI Study Group (2013). Ferric carboxymaltose prevents recurrence of anemia in patients with inflammatory bowel disease. Clin Gastroenterol Hepatol.

[18] Pels LP, Van de Vijver E, Waalkens HJ, Uitentuis J, Gonera-de Jong G, van Overbeek LA, Norbruis OF, Rings EH, van Rheenen PF (2010). Slow hematological recovery in children with IBD-associated anemia in cases of “expectant management”. J Pediatr Gastroenterol Nutr.

[19] Kulnigg-Dabsch S, Evstatiev R, Dejaco C, Gasche C (2012). Effect of iron therapy on platelet counts in patients with inflammatory bowel disease-associated anemia. PLoS One.

[CR20] The pre-publication history for this paper can be accessed here:http://www.biomedcentral.com/1471-230X/14/184/prepub

