# Supplementary material for: Effectiveness and safety of ferric carboxymaltose treatment in children and adolescents with inflammatory bowel disease and other gastrointestinal diseases
Source: BMC Gastroenterol. 2014 Oct 17;14:184. doi: 10.1186/1471-230X-14-184 (PMC4286929; doi:10.1186/1471-230X-14-184)
Supplement: Supplementary file 1 — Additional file 1: Table S1: Reference ranges for blood tests - used in the study - at various ages. (PDF 54 KB) [file 12876_2014_1205_MOESM1_ESM.pdf]

**Additional file 1: Table S1**

**Reference ranges for blood tests - used in the study - at various ages.**

| **HEMOGLOBIN in g/dl** | |
|---|---|
| **Age** | **Reference range** |
| 2 months - 3 months | 9.2 - 15.0 |
| 3 months - 4 months | 9.7 - 12.7 |
| 4 months - 6 months | 10.1 - 12.9 |
| 6 months - 1 year | 10.5 - 12.9 |
| 1 year - 2 years | 10.8 - 12.7 |
| 2 years - 3 years | 10.8 - 12.7 |
| 3 years - 10 years | 11.1 - 14.3 |
| 10 years - 12 years | 11.9 - 14.7 |
| 12 years - 16 years | 12.7 - 16.7 |
| >18 years (male) | 13.8 - 19.5 |
| >18 years (female) | 11.9 - 17.2 |
| | |
| **ERYTHROCYTES in Tpt/l** | |
| **Age** | **Reference range** |
| 2 months - 3 months | 2.80 - 4.80 |
| 3 months - 4 months | 3.10 - 4.70 |
| 4 months - 6 months | 3.20 - 5.20 |
| 6 months - 1 year | 3.60 - 5.20 |
| 1 year - 3 years | 3.70 - 5.30 |
| 3 years - 10 years | 3.70 - 5.70 |
| 10 years - 12 years | 3.80 - 5.80 |
| 12 years - 16 years | 4.30 - 5.90 |
| > 16 years (male) | 4.60 - 6.20 |
| > 16 years (female) | 4.20 - 5.40 |
| | |
| **MEAN CORPUSCULAR VOLUME in femtoliter (fL)** | |
| **Age** | **Reference range** |
| 2 months - 3 months | 81 - 121 |
| 3 months - 4 months | 77 - 113 |
| 4 months - 6 months | 73 - 109 |
| 6 months - 1 year | 74 - 102 |
| 1 year - 3 years | 73 - 101 |
| 3 years - 10 years | 72 - 88 |
| 10 years - 12 years | 69 - 93 |
| 12 years - 16 years | 72 - 90 |
| > 16 years | 80 - 96 |

| | |
|---|---|
| **HEMATOCRIT** | |
| **Age** | **Reference range** |
| 2 months - 3 months | 0.300- 0.440 |
| 3 months - 4 months | 0.310 - 0.430 |
| 4 months - 6 months | 0.320 - 0.440 |
| 6 months - 1 year | 0.350 - 0.430 |
| 1 year - 2 years | 0.350 - 0.430 |
| 2 years - 3 years | 0.350 - 0.430 |
| 3 years - 10 years | 0.310 - 0.430 |
| 10 years - 12 years | 0.330 - 0.450 |
| 12 years - 16 years | 0.360 - 0.480 |
| > 16 years (male) | 0.400 - 0.540 |
| > 16 years (female) | 0.370 - 0.470 |
| | |
| **SERUM FERRITIN LEVEL in µg/L** | |
| **Age** | **Reference range** |
| <= 1 month | 150.0 - 450.0 |
| 1 month - 3 months | 80.0 - 500.0 |
| 3 months - 18 years | 20.0 - 200.0 |
| | |
| **SOLUBLE TRANSFERRIN RECEPTOR in mg/L**: | |
| **Age** | **Reference range** |
| 1 month - 18 years | 0.76 - 1.76 |
| | |
| **SOLUBLE TRANSFERRIN RECEPTOR/ LOG FERRITIN RATIO** | |
| **Age** | **Reference range** |
| 1 month - 18 years | 0.38 - 1.54 |
| | |
| **TRANSFERRIN SATURATION in %** | |
| **Age** | **Reference range** |
| 1 month – 5 years | 7.0 - 44.0 |
| 5 years - 9 years | 17.0 - 42.0 |
| <= 14 years | 11.0 - 36.0 |
| <= 19 years | 6.0 - 33.0 |
| > 19 years | 16.0 - 45.0 |
